# Supplementary material for: Bronchial epithelial transcriptome reveals dysregulated interferon and inflammatory responses to rhinovirus in exacerbation-prone pediatric asthma
Source: JCI Insight. 2025 Nov 11;10(24):e197711. doi: 10.1172/jci.insight.197711 (PMC12890530; doi:10.1172/jci.insight.197711)
Supplement: Supplemental data [file jciinsight-10-197711-s172.pdf]

Transcriptomic Profiling of Bronchial Epithelium Reveals Dysregulated Interferon and Inflammatory Responses to Rhinovirus in Exacerbation-Prone Pediatric Asthma

Authors: Naresh Doni Jayavelu[1*], Basilin Benson[1*], Patricia C. dela Cruz[3], Weston T. Powell[2, 3], Lucille M. Rich[3], Elizabeth R. Vanderwall[3], Camile R. Gates[3], Andrew J Nagel[4], Maria P. White[3], Nyssa B. Samanas[3], Kourtnie Whitfield[5], Teal S. Hallstrand[6], Steven F. Ziegler[1], Matthew C. Altman[1, 4], Jason S. Debley[2,3**#]**

Supplemental material

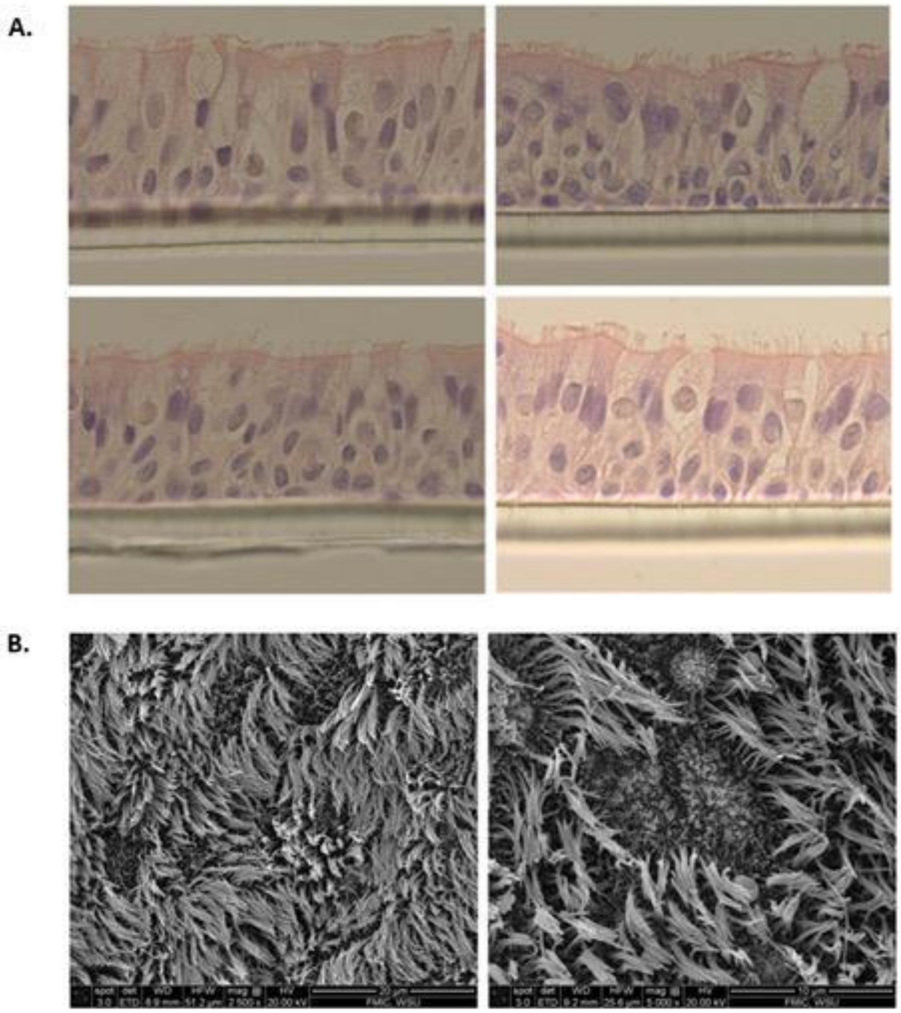

Supplemental Figure 1. Organotypic bronchial epithelial cultures. (**A**) Representative hematoxylin and eosin (H&E) stained histology cross sections and (**B**) scanning electron microscopy images of passage 3 primary bronchial epithelial cultures from study asthma donors differentiated to an organotypic pseudostratified and ciliated state for 21 days under air-liquid interface conditions using PneumaCult™ALI medium (Stemcell™).

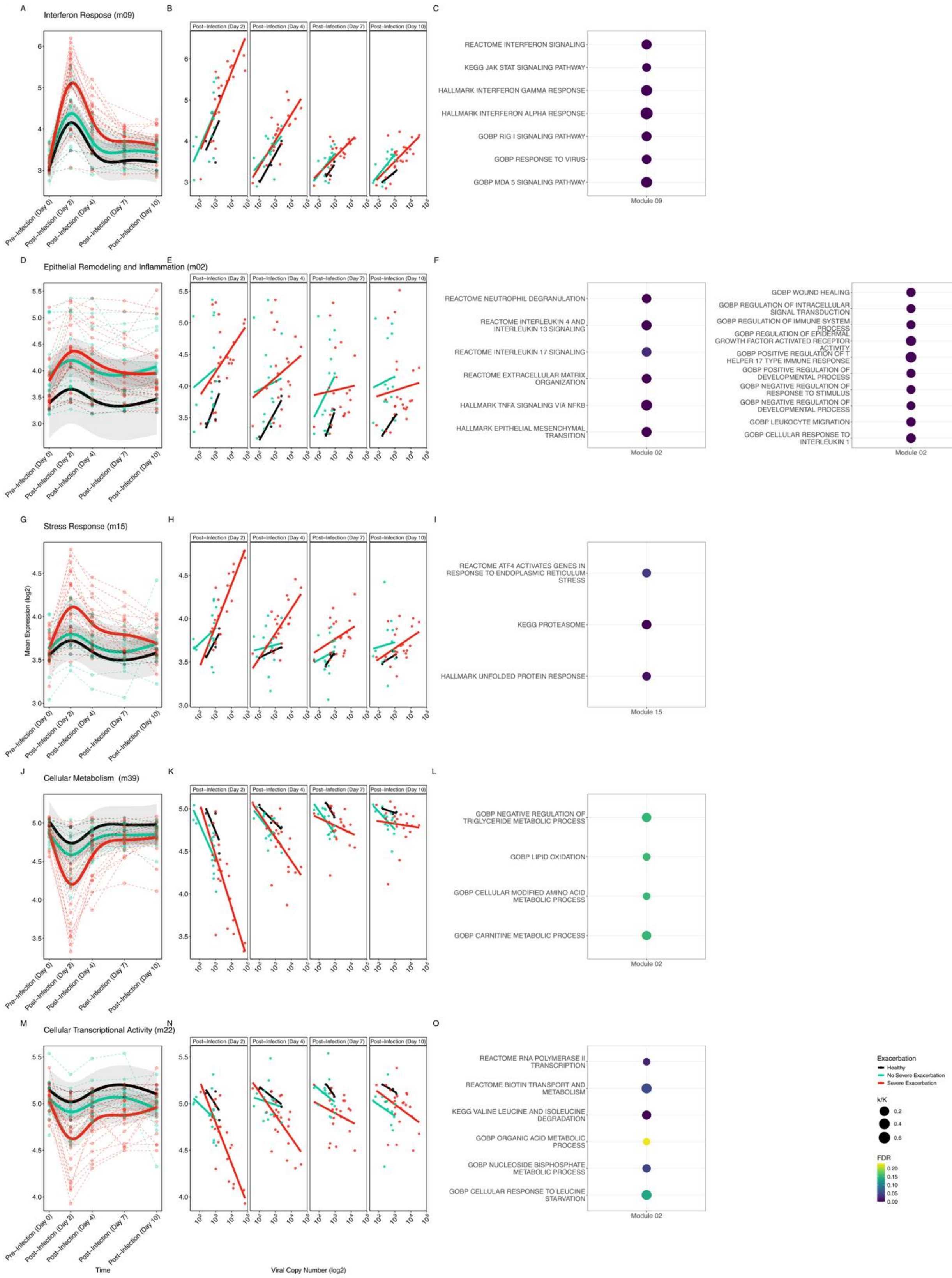

A
Interferon Respose (m09)
B
C
REACTOME INTERFERON SIGNALING
KEGG JAK STAT SIGNALING PATHWAY
HALLMARK INTERFERON GAMMA RESPONSE
HALLMARK INTERFERON ALPHA RESPONSE
GOBP RIG I SIGNALING PATHWAY
GOBP RESPONSE TO VIRUS
GOBP MDA 5 SIGNALING PATHWAY
Module 09
D
Epithelial Remodeling and Inflammation (m02)
E
F
REACTOME NEUTROPHIL DEGRANULATION
REACTOME INTERLEUKIN 4 AND INTERLEUKIN 13 SIGNALING
REACTOME INTERLEUKIN 17 SIGNALING
REACTOME EXTRACELLULAR MATRIX ORGANIZATION
HALLMARK TNFA SIGNALING VIA NFKB
HALLMARK EPITHELIAL MESENCHYMAL TRANSITION
Module 02
GOBP WOUND HEALING
GOBP REGULATION OF INTRACELLULAR SIGNAL TRANSDUCTION
GOBP REGULATION OF IMMUNE SYSTEM PROCESS
GOBP REGULATION OF EPIDERMAL GROWTH FACTOR ACTIVATED RECEPTOR ACTIVITY
GOBP POSITIVE REGULATION OF T HELPER 17 TYPE IMMUNE RESPONSE
GOBP POSITIVE REGULATION OF DEVELOPMENTAL PROCESS
GOBP NEGATIVE REGULATION OF RESPONSE TO STIMULUS
GOBP NEGATIVE REGULATION OF DEVELOPMENTAL PROCESS
GOBP LEUKOCYTE MIGRATION
GOBP CELLULAR RESPONSE TO INTERLEUKIN 1
Module 02
G
Stress Response (m15)
H
I
REACTOME ATF4 ACTIVATES GENES IN RESPONSE TO ENDOPLASMIC RETICULUM STRESS
KEGG PROTEASOME
HALLMARK UNFOLDED PROTEIN RESPONSE
Module 15
Mean Expression (log2)
J
Cellular Metabolism (m39)
K
L
GOBP NEGATIVE REGULATION OF TRIGLYCERIDE METABOLIC PROCESS
GOBP LIPID OXIDATION
GOBP CELLULAR MODIFIED AMINO ACID METABOLIC PROCESS
GOBP CARNITINE METABOLIC PROCESS
Module 02
M
Cellular Transcriptional Activity (m22)
N
O
REACTOME RNA POLYMERASE II TRANSCRIPTION
REACTOME BIOTIN TRANSPORT AND METABOLISM
KEGG VALINE LEUCINE AND ISOLEUCINE DEGRADATION
GOBP ORGANIC ACID METABOLIC PROCESS
GOBP NUCLEOSIDE BISPHOSPHATE METABOLIC PROCESS
GOBP CELLULAR RESPONSE TO LEUCINE STARVATION
Module 02
Post−Infection (Day 2)
Post−Infection (Day 4)
Post−Infection (Day 7)
Post−Infection (Day 10)
Pre−Infection (Day 0)
Time
Viral Copy Number (log2)
Exacerbation
Healthy
No Severe Exacerbation
Severe Exacerbation
k/K
0.2
0.4
0.6
FDR
0.20
0.15
0.10
0.05
0.00

Supplemental Figure 2. Time course of Module Expression and Correlation with Viral Load, with Functional Enrichment Annotations (**A, B**) Scatterplots showing the non-linear changes in expression of the "Interferon Response" module over time by donor group. BECs from SE Asthma donors exhibited a significantly higher average expression level at 2 days, with a 1.69-fold increase compared to NSE Asthma donors and a 1.92-fold increase compared to Healthy donors with a significantly different GAMM shape fit characterized by sustained higher levels at each subsequent timepoint (SE vs NSE: GAMM All timepoint: FDR=3.48e-05, linear model effect size restricted to d2: Estimate=0.76, FDR=9.47e-03, SE vs Healthy: GAMM All timepoint: FDR=9.87e-07, linear model effect size restricted to d2: Estimate=0.94, FDR=1.35e-1). (**C**) Selected enriched GO, Biocarta, Reactome, MSigDB Hallmark and KEGG terms for the 363 genes within the "Interferon Response" module. Dot size denotes the k/K value (ratio of number of genes in module (k) divided by the number of genes in the indicated geneset (K)), and the color denotes statistical significance of the pathway enrichment at FDR ranging from 0-0.20. (**D, E**) Analogous plots of the "Epithelial Remodeling and Inflammation" module. BECs from SE Asthma donors demonstrated a 1.15-fold higher expression level at 2d compared to NSE donors and sustained elevation at 4d with return to baseline levels in the NSE asthma group (SE vs NSE: GAMM All timepoint: FDR=8.4e-03, linear model effect size restricted to d2: Estimate=0.20, FDR=6.50e-01), and SE Asthma donors demonstrated a sustained greater expression compared to Healthy donors at each timepoint with a 1.67-fold higher expression at 2d (SE vs Healthy: GAMM All timepoint: FDR=6.55e-03, linear model effect size restricted to d2: Estimate=0.74, FDR=2.87e-01). (**F**) Selected enriched GO, Biocarta, Reactome, MSigDB Hallmark and KEGG terms for the 1396 genes within the "Epithelial

Remodeling and Inflammation" module. Dot size denotes the k/K value (ratio of number of genes in module (k) divided by the number of genes in the indicated geneset (K)), and the color denotes statistical significance of the pathway enrichment at FDR ranging from 0-0.20. (**G, H**) Analogous plots of the “Stress Response” module. BECs from SE Asthma donors demonstrated a 1.23-fold higher expression level at 2d compared to NSE donors and a 1.33-fold higher expression compared to Healthy donors, with sustained higher levels over time (SE vs NSE: GAMM All timepoint: FDR=1.8e-3, linear model effect size restricted to d2: Estimate=0.30, FDR=1.66e-02, SE vs Healthy: GAMM All timepoint: FDR=7.73e-04, linear model effect size restricted to d2: Estimate=0.41, FDR=1.53e-01). (**I**) Selected enriched GO, Biocarta, Reactome, MSigDB Hallmark and KEGG terms for the 242 genes within the “Stress Response" module. Dot size denotes the k/K value (ratio of number of genes in module (k) divided by the number of genes in the indicated geneset (K)), and the color denotes statistical significance of the pathway enrichment at FDR ranging from 0-0.20. (**J, K**) Scatterplot showing the non-linear changes in expression of the “Cellular Metabolism” module over time differing by clinical exacerbation group. BECs from SE Asthma donors demonstrated a significantly lower average expression level at 2 days, with a 1.30-fold decrease compared to NSE Asthma donors and a 1.52-fold decrease compared to Healthy donors with a significantly different GAMM shape fit characterized by sustained lower levels at each subsequent timepoint (SE vs NSE: GAMM All timepoint:FDR=8.1e-04, linear model effect size restricted to d2: Estimate=-0.38, FDR=1.66e-02, SE vs Healthy: GAMM All timepoint: FDR=9.80e-05, linear model effect size restricted to d2: Estimate=-0.60, FDR=1.12e-01). (**L**) Selected enriched GO, Biocarta, Reactome, MSigDB Hallmark and KEGG terms for the 106 genes within the “Cellular Metabolism"

module. Dot size denotes the k/K value (ratio of number of genes in module (k) divided by the number of genes in the indicated geneset (K)), and the color denotes statistical significance of the pathway enrichment at FDR ranging from 0-0.20. (**M, N**) Analogous plots of the "Cellular Transcriptional Activity" module. BECs from SE donors demonstrated a significantly lower average expression level at 2 days, with a 1.21-fold decrease compared to NSE donors and a 1.32-fold decrease compared to Healthy donors with a significantly different GAMM shape fit characterized by sustained lower levels at each subsequent timepoint (SE vs NSE: GAMM All timepoint:FDR =6.8e-04, linear model effect size restricted to d2: Estimate=-0.28, FDR=1.66e-02, SE vs Healthy: GAMM All timepoint: FDR=2.40e-03, linear model effect size restricted to d2: Estimate=-0.41, FDR=1.35e-01). (**O**) Selected enriched GO, Biocarta, Reactome, MSigDB Hallmark and KEGG terms for the 204 genes within the "Cellular Transcriptional Activity" module. Dot size denotes the k/K value (ratio of number of genes in module (k) divided by the number of genes in the indicated geneset (K)), and the color denotes statistical significance of the pathway enrichment at FDR ranging from 0-0.20.

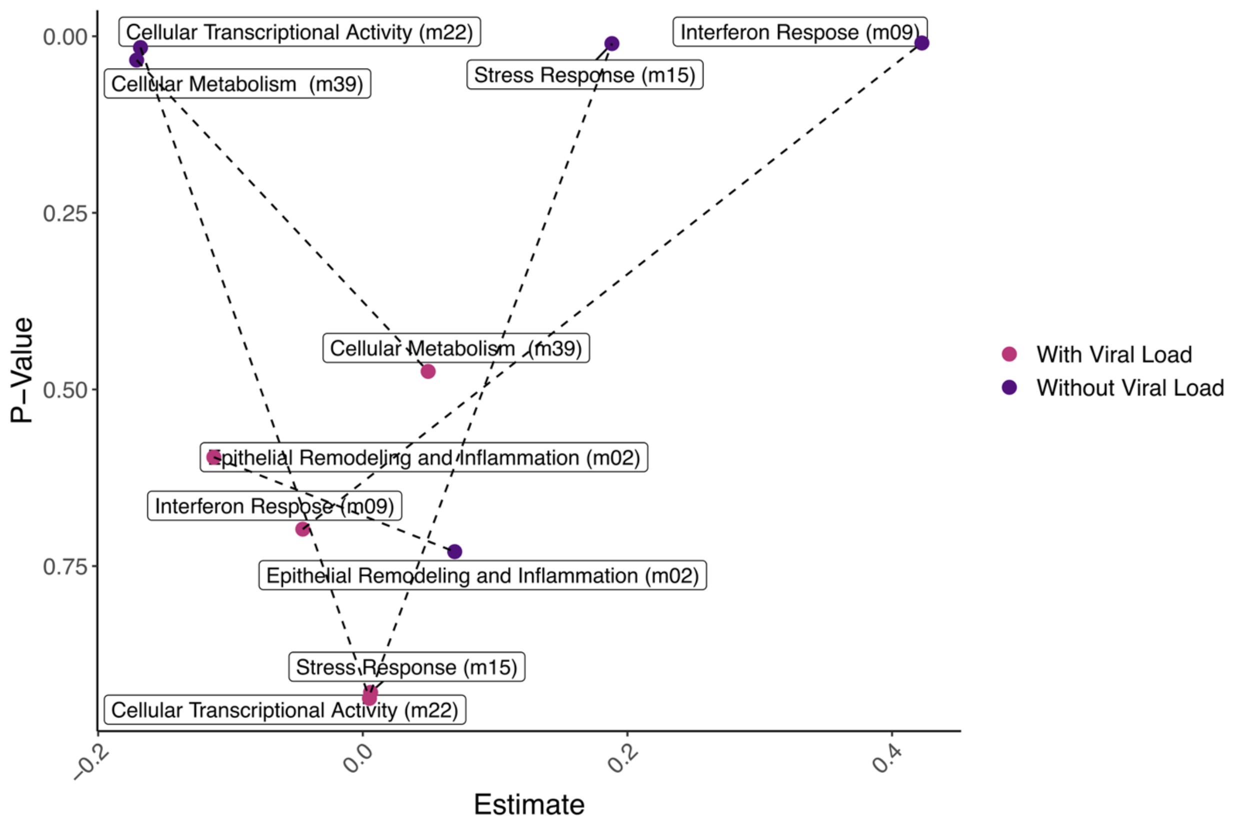

Supplemental Figure 3. Viral load has a mediating effect on Module expression. A linear model subset to the post infection timepoints, d2-10, was used to assess the extent to which viral load differences were mediating the magnitude differences in module expression observed between SE and NSE groups. Four of the modules of interest, Interferon response, Stress response, Cellular metabolism, and Cellular transcriptional activity, showed that viral load mediated the majority of difference in module expression observed between the two groups. The differences between SE and NSE in the Interferon response module were: without viral load: Estimate=0.42, p=9.56e-03; with viral load: Estimate=-0.045, p=0.69. Stress response module: without viral load: Estimate=0.14, p=0.024; with viral load: Estimate= -0.0014, p=0.98. Cellular metabolism module: without viral load: Estimate=-0.13, p=0.058; with viral load:

Estimate=0.024, p=0.66. Cellular transcriptional activity module: without viral load: estimate=-0.13, p=0.028; with viral load: Estimate=0.0033, p=0.95. No result could be derived for the Epithelial Remodeling and Inflammation module as this module largely differed by kinetic shape between groups rather than overall magnitude of expression Shown are the model estimates on x-axis and p-values on the y-axis for the model without (purple) or with (pink) viral load included in the model. The 5 modules of interest are labelled.

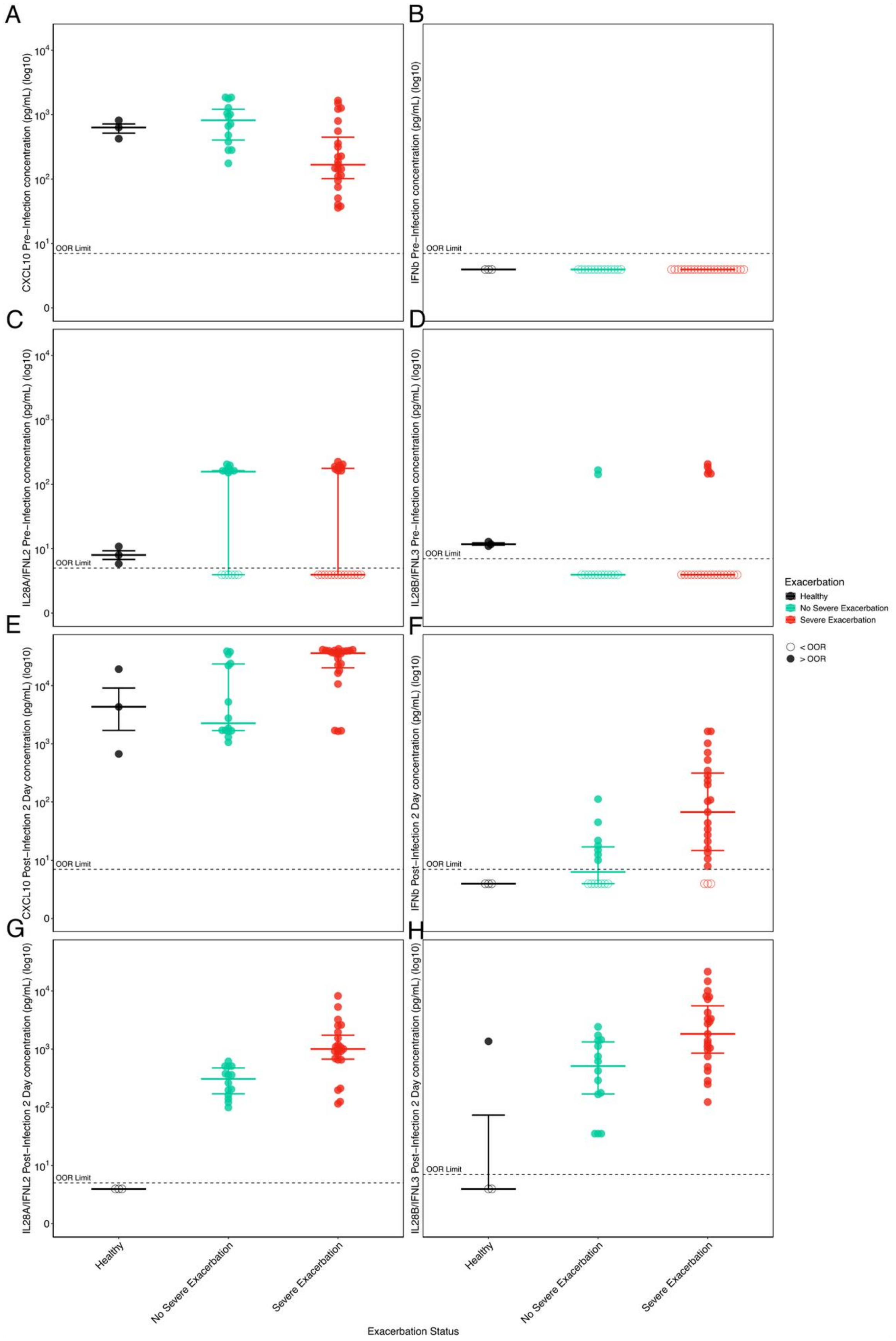

A
B
C
D
E
F
G
H
CXCL10 Pre−Infection concentration (pg/mL) (log10)
IFNb Pre−Infection concentration (pg/mL) (log10)
IL28A/IFNL2 Pre−Infection concentration (pg/mL) (log10)
IL28B/IFNL3 Pre−Infection concentration (pg/mL) (log10)
CXCL10 Post−Infection 2 Day concentration (pg/mL) (log10)
IFNb Post−Infection 2 Day concentration (pg/mL) (log10)
IL28A/IFNL2 Post−Infection 2 Day concentration (pg/mL) (log10)
IL28B/IFNL3 Post−Infection 2 Day concentration (pg/mL) (log10)
OOR Limit
Healthy
No Severe Exacerbation
Severe Exacerbation
Exacerbation Status
Exacerbation
< OOR
> OOR

Supplemental Figure 4. **Protein values by Exacerbation status** Dot plot showing log transformed pre- and post-infection *IFNB, IFNL2, IFNL3,* and *CXCL10* protein values by the Exacerbation groups and Healthy donors at 2 Day, boxplots indicate median and interquartile ranges for each group. Values that were below the detection limit are indicated as “OOR” (out of range).

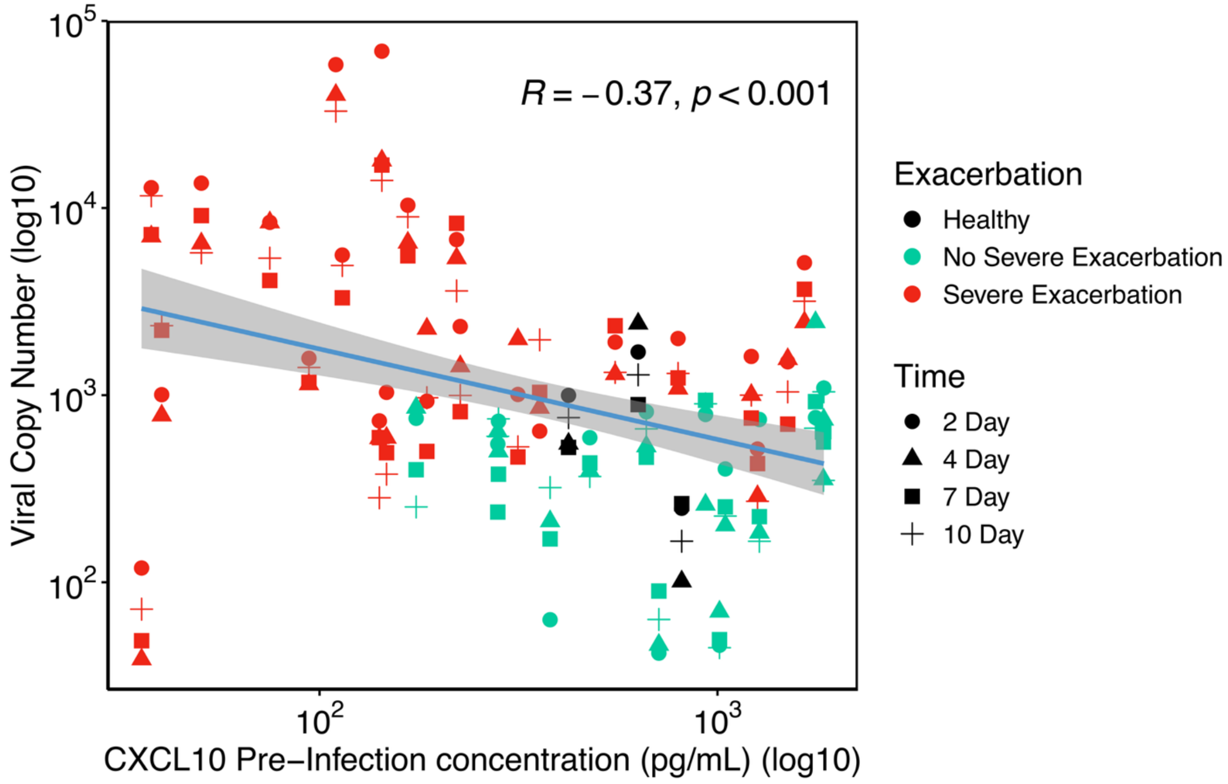

Supplemental Figure 5. Post-Infection Viral load shows a significant inverse relationship to Pre-Infection CXCL10. Scatter plot showing a significant inverse relationship between log transformed pre-infection CXCL10 protein secretion and log transformed viral load post-infection d2-d10.

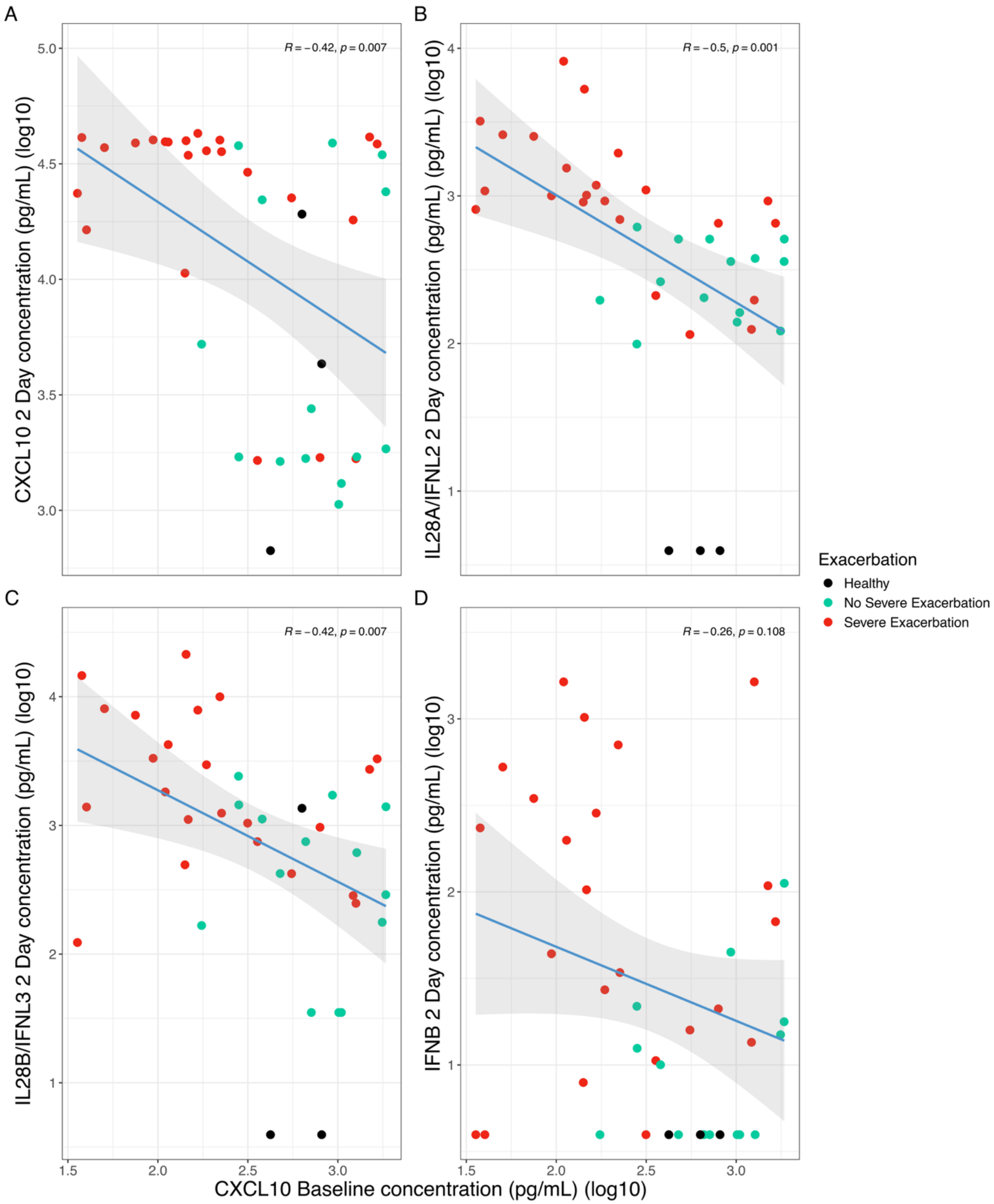

A
B
C
D
R = −0.42, p = 0.007
R = −0.5, p = 0.001
R = −0.42, p = 0.007
R = −0.26, p = 0.108
CXCL10 2 Day concentration (pg/mL) (log10)
IL28A/IFNL2 2 Day concentration (pg/mL) (log10)
IL28B/IFNL3 2 Day concentration (pg/mL) (log10)
IFNB 2 Day concentration (pg/mL) (log10)
CXCL10 Baseline concentration (pg/mL) (log10)
Exacerbation
Healthy
No Severe Exacerbation
Severe Exacerbation

Supplemental Figure 6. Pre-Infection CXCL10 shows a significant inverse relationship to Day 2 CXCL10, IFNβ, IFN-λ2/IL-28A and IFN-λ3/IL-28B. Scatter plot showing a significant inverse relationship between log transformed pre-infection CXCL10 protein secretion and subsequent protein secretion at 2 days post RV infection of (**A**) CXCL10 (Estimate=-0.37, p=7.16e-03), (**B**) IFN-λ2 (Estimate=-0.69, p=2.32e-05), and, (**C**) IFN-λ3 (Estimate=-0.34, p=4.24e-03) as well as a non-significant inverse trend with (**D**) IFN-β (Estimate=-0.14, p=1.69e-01). Fit lines are based on a linear model including 95% confidence intervals.

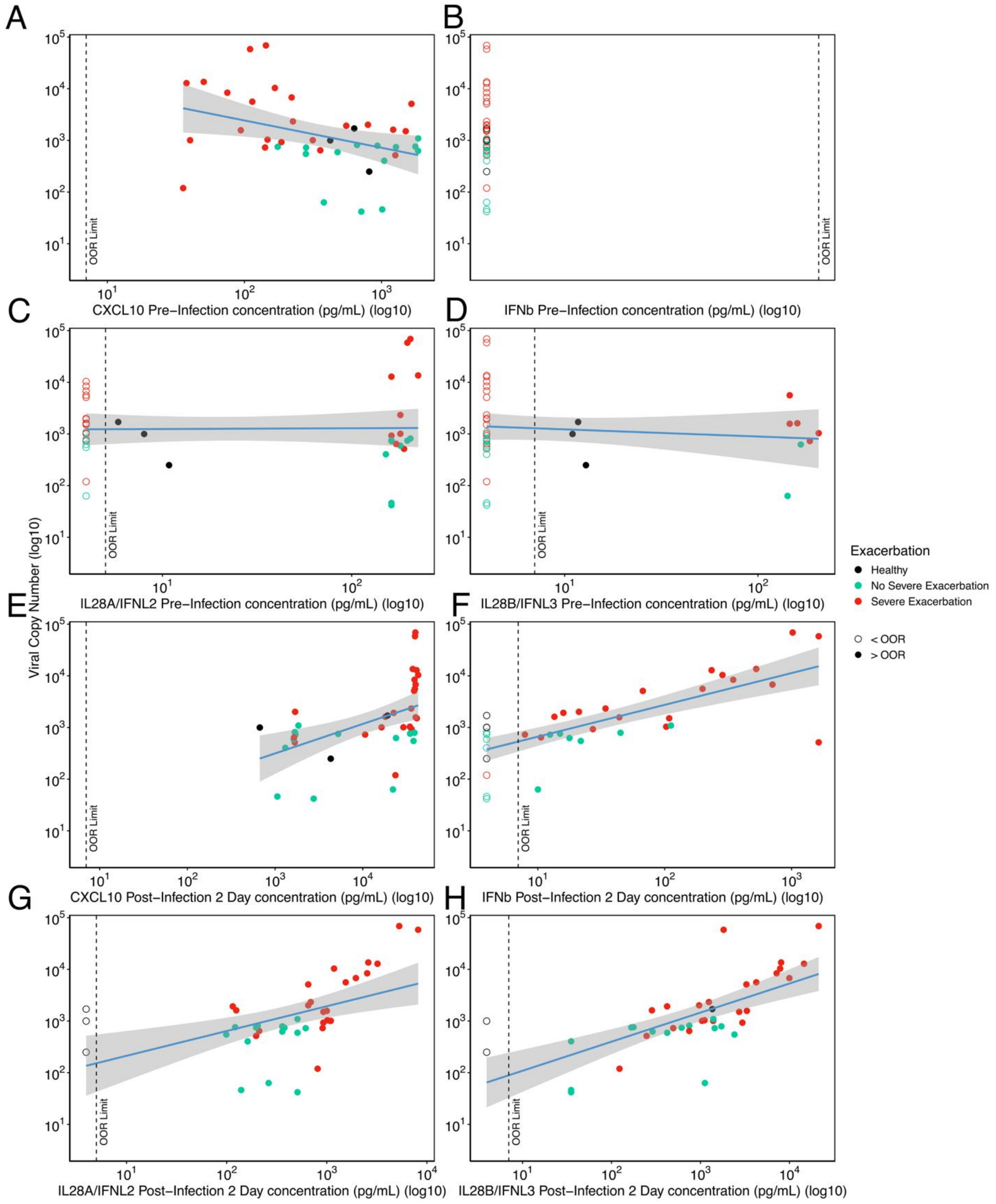

Supplemental Figure 7. Protein Correlations to viral copy number Scatterplots of pre- and post-infection *IFNB, IFNL2, IFNL3* and *CXCL10* protein values against viral

copy number values at 2 Day. Values that were below the detection limit are indicated as “OOR” (out of range)

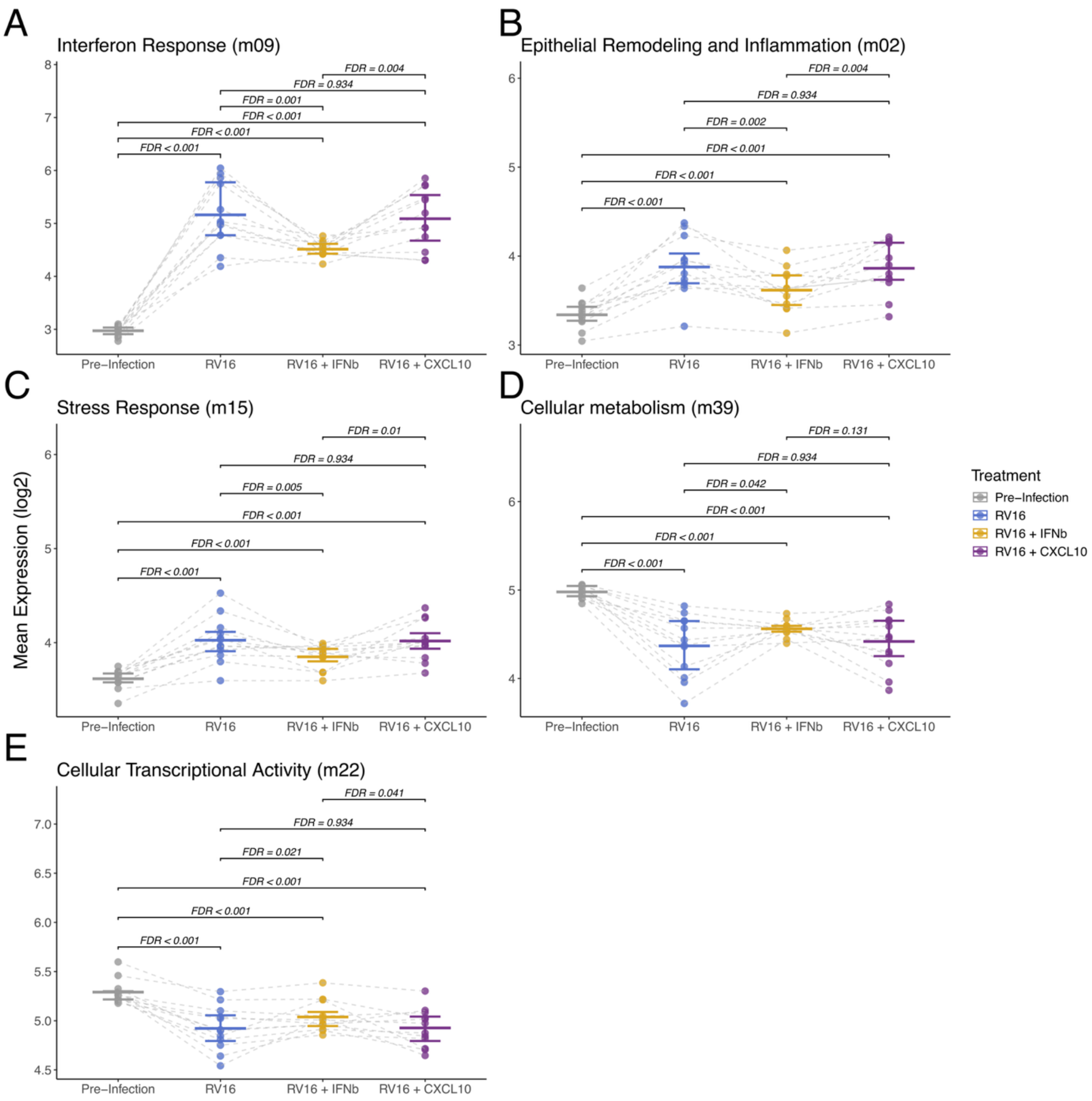

Supplemental Figure 8. Module Expression Difference by RV16, RV16 + IFN-β and RV16 + CXCL10 (**A**) Dot plot showing expression changes in Interferon response module by IFN-β treatment showing a 1.53-fold decrease in expression with IFN-β treatment compared to RV16 (estimate=-0.6, p=1.23e-03) and whereas there is a 4.42-fold and a 2.88-fold increase in expression from Pre-infection compared to RV16 infected sample and IFN-β treated samples respectively (RV16:estimate=2.14, p=2.74e-

15, IFN-β:estimate=1.52, p=4.25e-11). (**B**) Dot plot showing expression changes in Epithelial remodelling and Inflammation response module by IFN-β treatment showing a 1.20-fold decrease in expression with IFN-β treatment (estimate=-0.27, p=2.24e-03) and whereas there is a 1.47-fold and a 1.21-fold increase in expression from Pre-infection compared to RV16 infected sample and IFN-β treated samples respectively (RV16:estimate=0.55, p=9.41e-09, IFN-β:estimate=0.28, p=3.23e-04). (**C**) Dot plot showing expression changes in Stress response module by IFN-β treatment showing a 1.14-fold decrease in expression with IFN-β treatment (estimate=-0.19, p=4.61e-03) and whereas there is a 1.35-fold and a 1.18-fold increase in expression from Pre-infection compared to RV16 infected sample and IFN-β treated samples respectively (RV16:estimate=0.43, p=1.39e-08, IFN-β:estimate=0.23, p=2.41e-04). (**D**) Dot plot showing expression changes in Cellular metabolism module by IFN-β treatment showing a 1.12-fold increase in expression with IFN-β treatment (estimate=0.16, p=4.20e-02) and whereas there is a 1.48-fold and a 1.32-fold decrease in expression from Pre-infection compared to RV16 infected sample and IFN-β treated samples respectively (RV16:estimate=-0.56, p=1.18e-08, IFN-β:estimate=-0.40, p=5.64e-06). (**E**) Dot plot showing expression changes in Cellular transcriptional activity module by IFN-β treatment showing a 1.09-fold increase in expression with IFN-β treatment (estimate=0.13, p=2.13e-02) and whereas there is a 1.31-fold and a 1.19-fold decrease in expression from Pre-infection compared to RV16 infected sample and IFN-β treated samples respectively (RV16:estimate=-0.39, p=1.18e-08, IFN-β:estimate=-0.25, p=1.57e-05) . Boxplots indicate median and interquartile ranges for each group and the Fit line is based on a linear model including 95% confidence intervals.

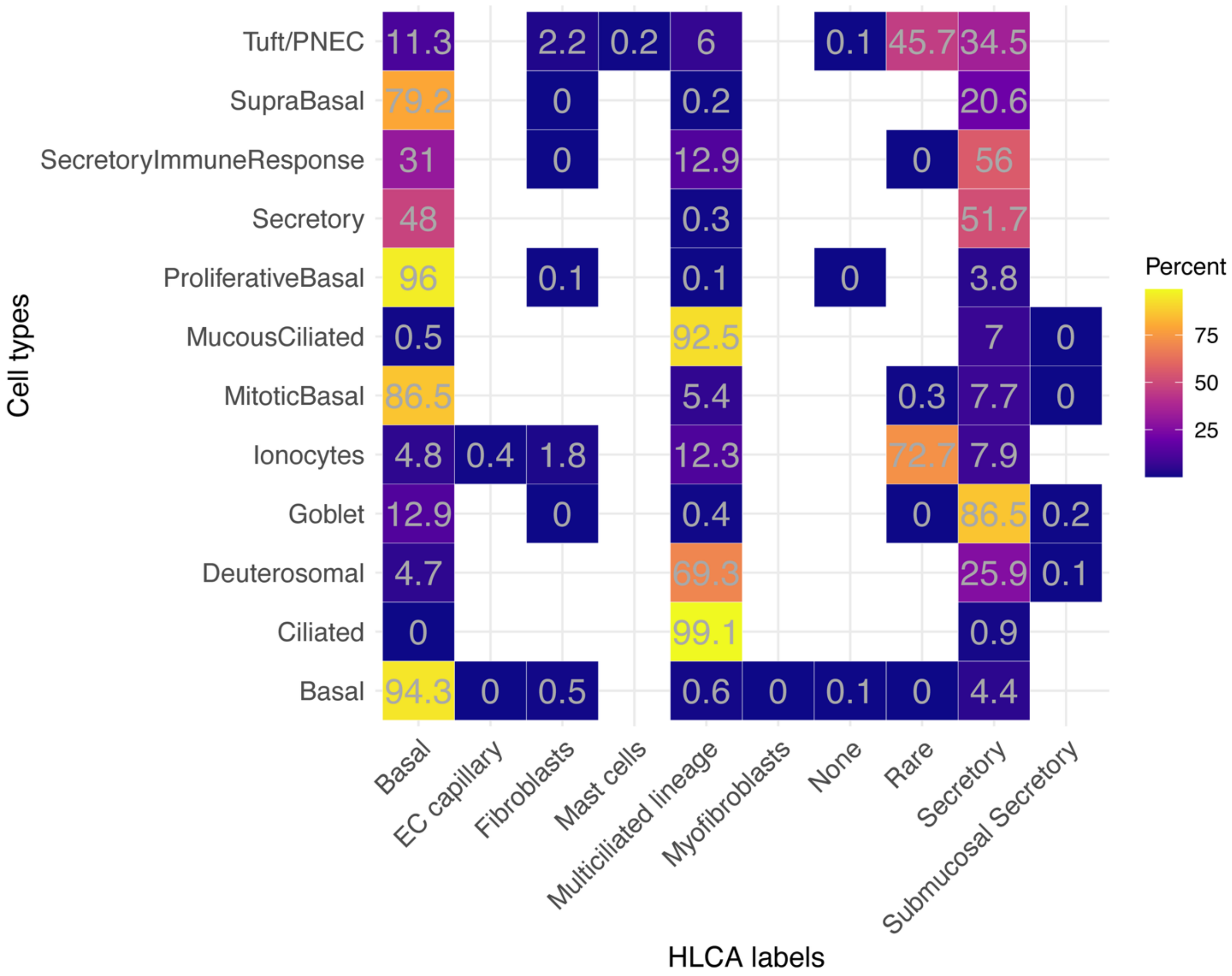

Supplemental Figure 9. Clusters mapping to HLCA cell type labels. Heatmap showing the fraction of cells from our annotated cell types that correspond to one or more Human Lung Cell Atlas (HLCA) labels. Color intensity represents the percentage of overlap, with actual percents indicated within each tile.

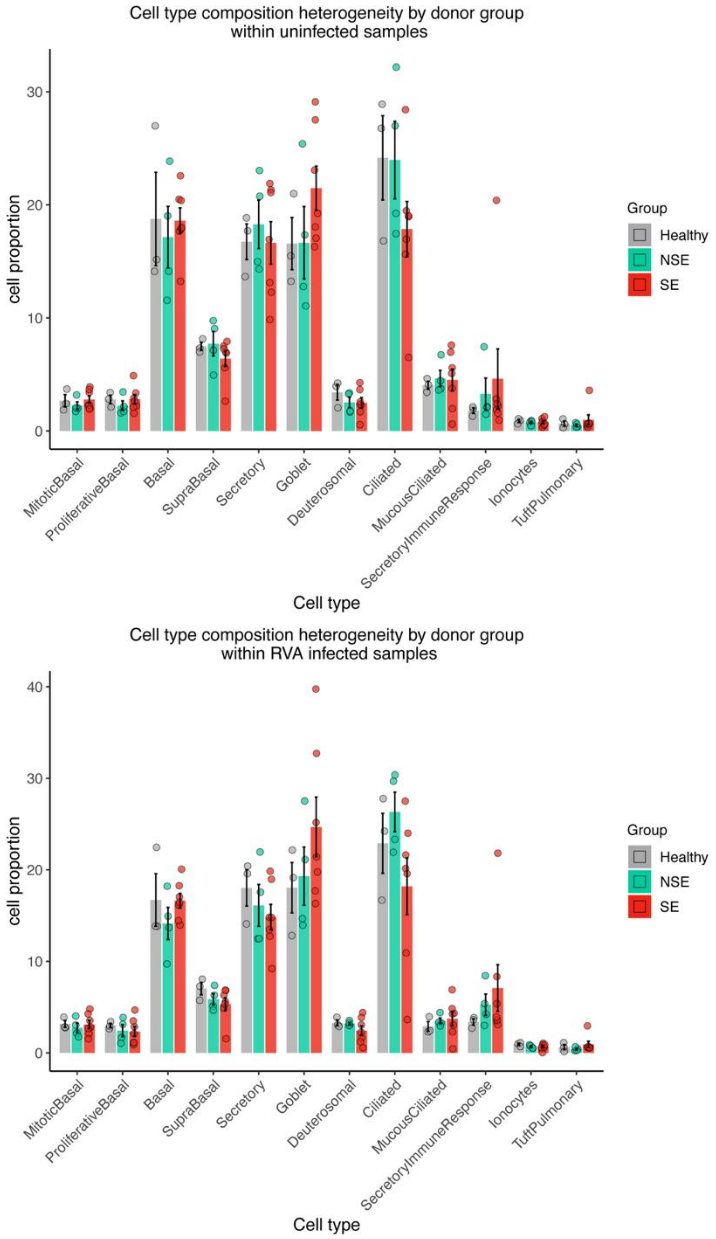

Supplemental Figure 10. Cell compositional differences: Bar plots showing the cell compositional levels (in percent) across donor groups (Healthy, NSE, SE asthma) in uninfected samples displayed on the top panel and samples collected 2 days post RV infection on the bottom panel. The error bars denote the variability across donors and circles represent individual donor cell proportions.

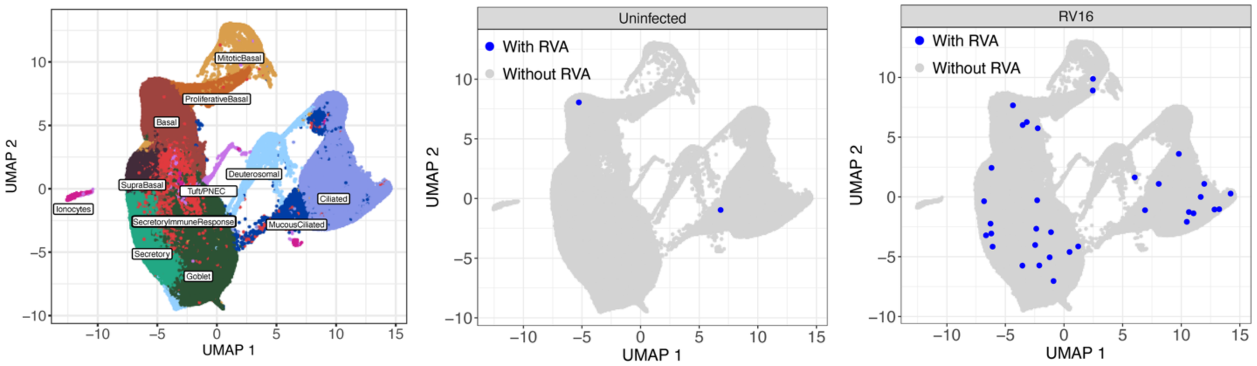

Supplemental Figure 11. Rhinovirus infected cells: UMAP with annotated cell types on the left panel and an overlay of infected (blue colored points) vs uninfected cells (gray colored points) on the middle (Uninfected samples) and right panels (RV16 infected samples).

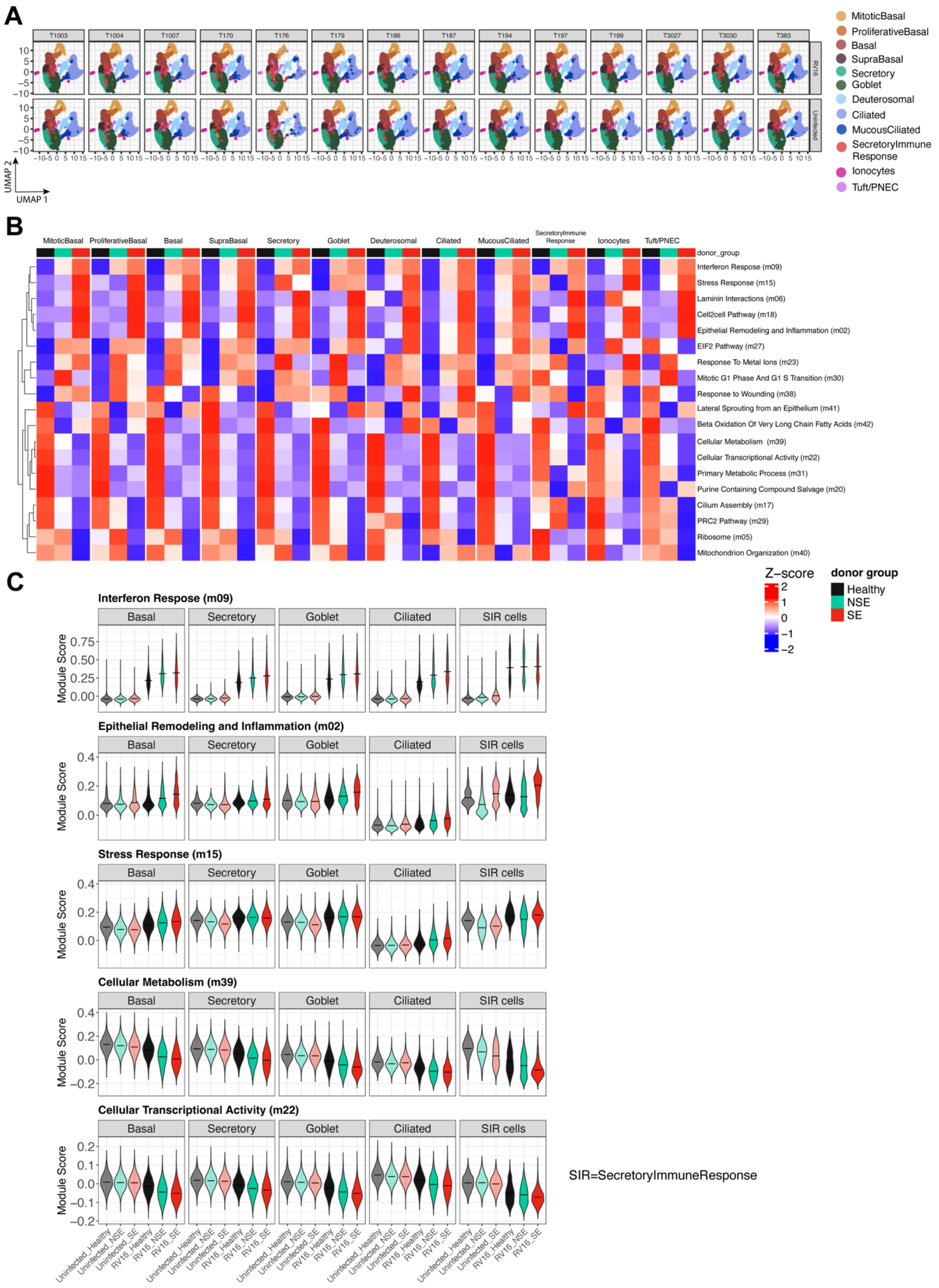
A
T1003
T1004
T1007
T170
T176
T179
T186
T187
T194
T197
T199
T3027
T3030
T383
RV16
Uninfected
UMAP 2
UMAP 1
MitoticBasal
ProliferativeBasal
Basal
SupraBasal
Secretory
Goblet
Deuterosomal
Ciliated
MucousCiliated
SecretoryImmune Response
Ionocytes
Tuft/PNEC
B
donor_group
Interferon Respose (m09)
Stress Response (m15)
Laminin Interactions (m06)
Cell2cell Pathway (m18)
Epithelial Remodeling and Inflammation (m02)
EIF2 Pathway (m27)
Response To Metal Ions (m23)
Mitotic G1 Phase And G1 S Transition (m30)
Response to Wounding (m38)
Lateral Sprouting from an Epithelium (m41)
Beta Oxidation Of Very Long Chain Fatty Acids (m42)
Cellular Metabolism (m39)
Cellular Transcriptional Activity (m22)
Primary Metabolic Process (m31)
Purine Containing Compound Salvage (m20)
Cilium Assembly (m17)
PRC2 Pathway (m29)
Ribosome (m05)
Mitochondrion Organization (m40)
Z–score
donor group
Healthy
NSE
SE
C
Interferon Respose (m09)
Epithelial Remodeling and Inflammation (m02)
Stress Response (m15)
Cellular Metabolism (m39)
Cellular Transcriptional Activity (m22)
Basal
Secretory
Goblet
Ciliated
SIR cells
Module Score
Uninfected_Healthy
Uninfected_NSE
Uninfected_SE
RV16_Healthy
RV16_NSE
RV16_SE
SIR=SecretoryImmuneResponse

Supplemental Figure 12. Single cell transcriptomics, module level analysis. (**A**) UMAP representation of the 316,712 cells separated by donor and condition (uninfected and RV infection). (**B**) Heatmap showing relative expression of modules (defined by WGCNA from bulk transcriptomics) expression at 48 hr following RV infection across donor groups, healthy, non-Severe and Severe at individual cell types . Module expression levels are shown as row normalized Z-scores of the mean expression for each group with red representing higher relative expression and blue representing lower relative expression. Module row ordering is by hierarchical clustering. (**C**) Violin plot showing distribution of module expression for select modules by condition (uninfected and RV infection) by donor groups, healthy, non-Severe and Severe at individual cell types. The black horizontal line in each violin denotes a mean expression.

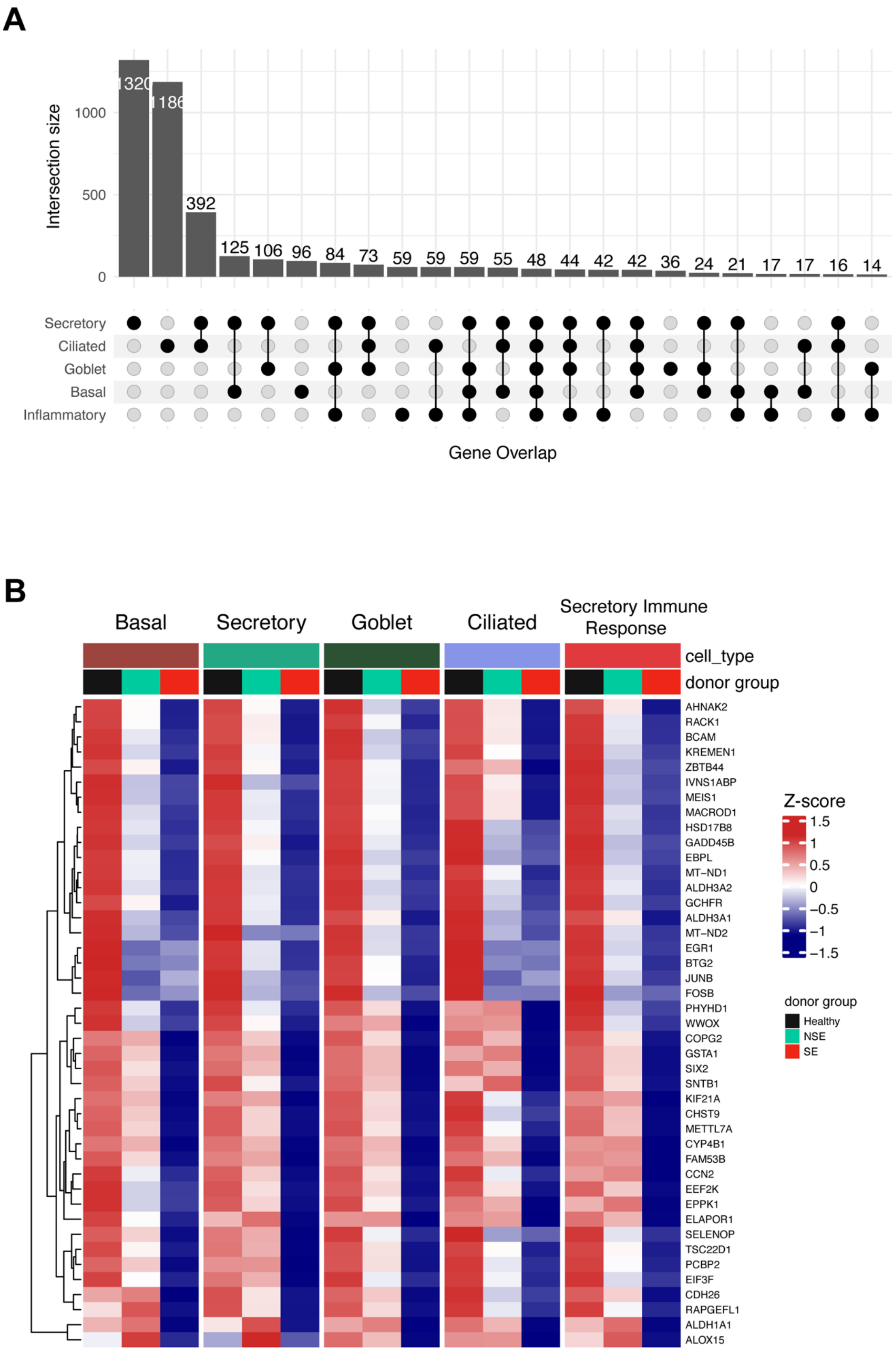
A
Intersection size
1000
500
0
1320
1186
392
125
106
96
84
73
59
59
59
55
48
44
42
42
36
24
21
17
17
16
14
Secretory
Ciliated
Goblet
Basal
Inflammatory
Gene Overlap
B
Basal
Secretory
Goblet
Ciliated
Secretory Immune Response
cell_type
donor group
AHNAK2
RACK1
BCAM
KREMEN1
ZBTB44
IVNS1ABP
MEIS1
MACROD1
HSD17B8
GADD45B
EBPL
MT−ND1
ALDH3A2
GCHFR
ALDH3A1
MT−ND2
EGR1
BTG2
JUNB
FOSB
PHYHD1
WWOX
COPG2
GSTA1
SIX2
SNTB1
KIF21A
CHST9
METTL7A
CYP4B1
FAM53B
CCN2
EEF2K
EPPK1
ELAPOR1
SELENOP
TSC22D1
PCBP2
EIF3F
CDH26
RAPGEFL1
ALDH1A1
ALOX15
Z-score
1.5
1
0.5
0
−0.5
−1
−1.5
donor group
Healthy
NSE
SE

Supplemental Figure 13. Single cell transcriptomics, gene level analysis. (**A**) Upset plot showing the count of overlapping genes with descending trend across five cell types, basal, secretory, goblet, ciliated and secretory immune response cells. (**B**) Heatmap showing relative expression of 48 core signature genes (shared across all five cell types) with descending expression trends at 5 cell types. Gene expression levels are shown as row normalized Z-scores of the mean expression for each group with red representing higher relative expression and blue representing lower relative expression.

Supplemental Tables

Supplemental Table 1. Generalized Additive Mixed Models (GAMMs) results comparing expression differences over time between healthy and the two exacerbation groups. Table shows the smoothing terms of time and smoothing interaction term for the effect of exacerbation groups and healthy samples over time and shows the average difference by the exacerbation and healthy groups.

Supplemental Table 2. Results of the Linear Model shows the differential expressed modules by viral load over time and subset to Day 2

Supplemental Table 3A: Module annotations based on gene enrichment
Supplemental Table 3B: Table of genes in Modules EnsembleIDs
Supplemental Table 3C: Table of genes in Modules hgnc symbol

Supplemental Table 4. Linear Model results comparing expression at Day 2 between healthy and the two exacerbation groups.

Supplemental Table 5. Linear mixed effects models results to check for mediation effect of viral load

Supplemental Table 6. Linear model comparison of baseline BEC supernatant concentrations by exacerbation group

Supplemental Table 7. Linear Model results comparing expression difference between RV16, IFNb and CXCL10 Treatment groups.

Supplemental Table 8. Linear Model comparison of viral copy number to the module expression.

Supplemental Table 9. Linear mixed effects models results to check for mediation effect of viral load with Treatment group RV16, RV16 + IFNb and RV16 + CXCL10

Supplemental Table 10. Cell Type-Specific Marker Genes. This table lists the top marker genes that distinguish each cell type from the others, as identified through differential expression analysis. These markers were used to define and validate cell type annotations across the dataset.

Supplemental Table 11. Cell Counts by Donor, Condition, and Cell Type. This table provides a breakdown of cell counts stratified by individual donor ID, donor group (healthy controls, NSE asthma, SE asthma), experimental condition (uninfected vs.RV-infected), and annotated cell type.

Supplemental Table 12. CLM Module-Level Estimates in RV-Infected Samples
Contains output from the Cumulative Linked Model (CLM) ordinal regression framework, reporting model estimates for all modules across all cell types within the RV-infected condition.

Supplemental Table 13. CLM Gene-Level Estimates in RV-Infected Samples
Contains output from the Cumulative Linked Model (CLM) ordinal regression framework, reporting model estimates for genes across all cell types within the RV-infected condition.

Supplemental Table 14. Enriched Biological Terms from CLM-Identified Genes
Lists significantly enriched biological pathways and gene ontology terms derived from the set of genes identified as significant in the CLM model under RV-infected conditions.
